# Comparative Evaluation of the *In Vitro* Anthelminthic Effects of the Leaves, Stem, and Seeds of *Carica papaya* (Linn) Using the *Pheretima posthuma* Model

**DOI:** 10.1155/2020/9717304

**Published:** 2020-05-17

**Authors:** Phoebe Esinam Goku, Emmanuel Orman, Anna Naa Kwarley Quartey, George Twum Ansong, Elsam Baffour Asare-Gyan

**Affiliations:** ^1^Department of Pharmaceutical Sciences, School of Pharmacy, Central University, Accra 23321, Ghana; ^2^Department of Pharmaceutical Chemistry, School of Pharmacy, University of Health and Allied Sciences, Ho 23321, Ghana

## Abstract

This study was conducted to comparatively assess the anthelminthic activity of leaves, stem bark, and seeds of *Carica papaya,* in order to identify which of the plant parts possess the highest anthelminthic activity. Three concentrations of ethanolic and hydroethanolic extracts of the plant parts (1 mg/ml, 2.5 mg/ml, and 5 mg/ml) were prepared and tested against *Pheretima posthuma* using albendazole as the positive control and 0.9% normal saline solution as the negative control. Preliminary phytochemical investigation showed the presence of alkaloids, saponins, and reducing sugars of glycosides present in all the crude extracts of *Carica papaya*. Tannins were observed only in extracts of the leaves, while fixed oils were only present in the extracts of the seeds. The results of the anthelminthic activity testing indicated that all crude extracts prepared were more effective than albendazole in reducing paralysis time (*p* < 0.0001) and death time (*p* < 0.0001). It was further shown that the extracts from the seeds (SE and SHE) were more effective than the extracts from the stem bark and leaves both in reducing paralysis and death times. Fractionation of SE provided a fraction, SE_B_, which was more effective than SE both in reducing paralysis and death times (*p* < 0.0001) and was established to contain fixed oils. The outcome of the current study has provided a scientific justification for the preference of the seeds of *Carica papaya* for the treatment of helminth infections and has shown that the fixed oils present in the seeds could be responsible for such activity.

## 1. Introduction

It is generally held that helminth infections are the most commonly encountered infections in man [1], with an estimation of approximately three million people infected globally [2]. When an individual is infected, the person excretes faeces infected with helminth eggs, thereby contaminating the soil [1]. Helminth infections usually result in cases of anaemia, pneumonia, eosinophilia, and malnutrition [1]. Other reported symptoms include abdominal pains, nausea, and diarrhoea [3]. Although the infections are not known to be lethal as compared to other infections, they are thought to be recurrent among poor people living in poor sanitary conditions where due to financial constraints are unable to access effective conventional medications [4], and for that reason pose an enormous impact economically on human livelihood [5].

Helminthiases present usually in two clinically important forms: the first consisting of organisms which reside in the host's gastrointestinal tract and the other including organisms which reside in other tissues of the host's body [1]. Intestinal hookworms (*Ancylostoma duodenale* and *Necator americanus*), roundworms (*Ascaris lumbricoides*), whipworms (*Trichuris trichiura*) [1, 3, 4], and tapeworms (*Taenia saginata, Taenia solium, Hymenolepis nana,* and *Diphyllobothrium latum*) usually reside in the host's gastrointestinal tract, while trematodes (*Schistosoma haematobium*, *Schistosoma mansoni*, and *Schistosoma japonicum*), tissue roundworms (*Trichinella spiralis* and *Dracunculus medinensis*), and hydatid tapeworms (*Echinococcus* sp.) reside in the host's tissues [1].

Over the years, conventional anthelminthic drugs, such as, albendazole, mebendazole, ivermectin, piperazine, and niclosamide, have been widely patronized to treat helminth infections, in both livestock and humans. However, due to factors such as cost of these medicines [6], their toxic effects [7], and reported cases of resistance development [5], their patronage and usefulness especially among people living in low-middle income countries have been limited and substituted with cheaper alternatives, mainly, herbal medicines [7]. Several studies have documented the traditional or folkloric use of herbal medicines for helminthiases [1, 4, 8], and scientific justifications have been widely documented for some of these medicinal plants [2, 7, 9–11].

The name *Carica papaya* is almost synonymous to plant-based anthelminthic agents. *Carica papaya Linn*. (Caricaceae) is well known for its nutritional and medicinal values. The various parts of the plant are used in different health conditions [12]. The leaves are thought to increase breast milk and used in the treatment of tonsillitis, ulcerative stomatitis, and gingivitis [12]. The roots have been suggested to be beneficial in managing arsenic poisoning [13]. Different preparations from the leaves have found use in managing haemorrhoids, management of asthma, treatment of urinary tract infections, poultice for sores [12], and treatment of helminth infections [14]. The fruits are used to treat indigestion [15], chronic diarrhoea, ringworm infections, bleeding piles, and amoebic dysentery [14]. The stem bark, flowers, roots, and seeds have all been well documented to be used for the management of several conditions, chiefly among them are their roles in managing helminth infections [4, 7, 14, 16].

In Ghana, a study conducted showed that 74% traditional healers used the plant for helminth infections [4]. A previous study conducted established *in vitro* anthelminthic activity for the leaves and the stems [16] against *Pheretima posthuma*. Another study established the *in vitro* activity of the seeds against intestinal worms, *Haemonchus contortus*, *Oesophagostomum* sp., *Trichostrongylus* sp., and *Cooperia* sp. [7]. It could be argued that due to differences in the chemical composition of the various parts of the plant, there may be observable differences in their anthelminthic activities. The current study, thus, sought to compare the activities of these parts and provide knowledge on the most effective part of the plant. The study further considered identifying which group of phytochemicals may be responsible for the activity.

## 2. Materials and Methods

### 2.1. Drugs, Chemicals, and Reagents

The reference drug adopted was albendazole (Kela N.V, Belgium, Man. Date: 4/01/2018; Exp. Date: 4/01/2020) procured from a retail community pharmacy in the Accra metropolis. All chemicals and reagents used were of analytical grade; they wereferric chloride powder, Dragendorff's reagent, 1% gelatin solution, Wagner's reagent, and Fehling's reagent (ReAgent Chemicals Company, Cheshire, England), sodium bicarbonate powder, hexane (40–60°C), and ethyl acetate solution (Infinite Fine Chemicals, London, England), hydrochloric acid solution and silica gel (Medved, Russia), sulphuric acid solution (Maalab Scientific Equipment PVT Limited, India), glacial acetic acid solution (Bide Pharmatech Ltd, India), ammonia solution (Pentagon Chemical Specialties Limited, United Kingdom), potassium hydroxide powder (Osnova Chemical Factory Limited, Ukraine), ethanol (98%), distilled water (in-house), iodine crystals (Fisher Scientific, UK), and normal saline (Sanbao Pharmaceuticals Limited, Accra, Ghana).

### 2.2. Glassware and Apparatus

Erlenmeyer's flasks, test tubes and rack, funnel, sieve, measuring cylinder, desiccator, dropper, plastic containers, petri dishes, TLC plates, chromatography columns, cotton wool, retort stand and clamp, cover plates, reagent bottles, filter paper, dropper, spatula, droppers, stirring rod, conical flask, beaker, stop watch, pencil, meter rule, and mortar and pestle were used at different stages in the study.

### 2.3. Instrumentation

Light microscope (Leica DM4 P, DM2700 P, Leica microsystems, Germany), analytical balance (HZ series, maximum range 3002 model), pH meter (Bench MV pH meter with 0.001 pH resolution, pH 213 model, Sigma Aldrich), UV spectrophotometer chamber (UV Chamber Pt1000 RTD model, DNG Technologies Private Limited, India), and rotary evaporator (RV 3 model, Thomas Scientific Limited, UK) were used in the study.

### 2.4. Collection and Preparation of Plant Materials

The leaves, stem bark, and seeds of *Carica papaya* Linn. (Caricaceae) were used for the study. These were collected on October 22, 2018, from a farm located at Krobo-Odumase in the Eastern Region of Ghana. They were then authenticated at the Department of Pharmacognosy, Central University, School of Pharmacy, with voucher specimen number kept at the school's herbarium. The voucher specimen numbers for the samples were as follows: seeds, *CARICA* Seeds/2018/01; leaves, C*ARICA* Leaves/2018/02, and stem, *CARICA* Stem/2018/03. The samples were thoroughly washed with distilled water, to get rid of unwanted foreign matter, cut into pieces, and dried separately under shade in a cool dry environment for four weeks. The dried samples were then milled into fine powder and stored in airtight plastic containers and labelled accordingly. The samples were later subjected to extraction, phytochemical analysis, anthelmintic activity investigation, and chromatography.

### 2.5. Test Organisms

**Figure 1:**
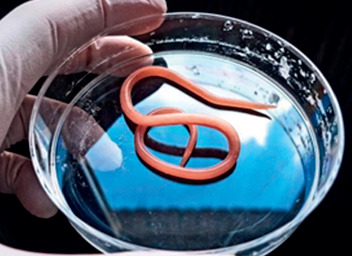
Picture of an adult earthworm, *Pheretima posthuma* (Pheritimidae).

Adult earthworms, *Pheretima posthuma* (Pheritimidae), were used for the study because of their anatomical and physiological resemblance with the human intestinal roundworm parasite, *Ascaris lumbricoides* [2, 5]. The organisms were collected from the moist soils of the biodiversity forest in Central University, Miotso in the Tema Metropolis, and washed with normal saline, to remove foreign and faecal matter. They were then authenticated in the Department of Anatomy, School of Medicine and Health Sciences, Central University. The selected earthworms were 5 to 7 cm in length and 0.1 to 0.3 cm in width (Figure 1).

### 2.6. Extraction

**Figure 2:**
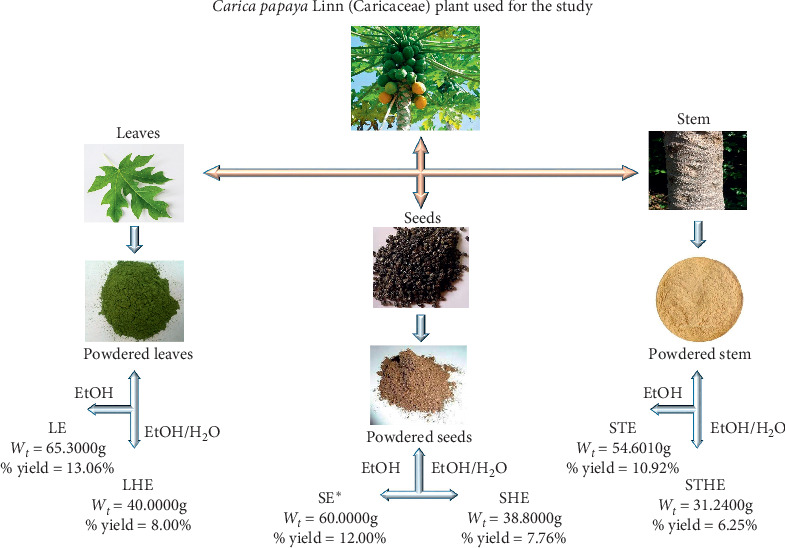
Scheme for extraction and preparation of the crude extracts. **∗**Most effective of the crude extracts. EtOH: outcome from ethanolic extraction; EtOH/H_2_O: outcome from hydroethanolic extraction; LE: crude ethanolic extract from leaves; LHE: crude hydroethanolic extract from leaves; SE: crude ethanolic extract from seeds, SHE: crude hydroethanolic extract from seeds, STE: crude ethanolic extract from stem, and STHE: crude hydroethanolic extract from stem.

For extraction, the powdered samples for each plant part were divided into two: the first in each case was extracted with ethanol (98%) while the second was extracted with a hydroalcoholic solvent (ethanol and water in the ratio 2 : 1). Plant samples were cold macerated for 72 hours, after which filtrates were concentrated *in vacuo*, packaged, and stored for later use (Figure 2).

### 2.7. Phytochemical Screening

The freshly prepared crude extracts from the leaves (LE and LHE), stem (STE and STHE), and seeds (SE and SHE) of *Carica papaya* were subjected to standard qualitative phytochemical screening tests for the following secondary metabolites: tannins, alkaloids, fixed oils, reducing sugars of glycosides, and saponins according to methods described in the literature [17–19].

### 2.8. Preparation of Solutions

1 mg/ml, 2.5 mg/ml, and 5.0 mg/ml concentrations of the crude extracts obtained (Figure 2) were prepared by dissolving specified quantities of the extracts in normal saline. Similar concentrations were also prepared from the albendazole tablets by suspending specified quantities of the crushed tablets in normal saline.

### 2.9. *In Vitro* Anthelminthic Effects of Crude Extracts

The method adopted was as described in literature with modifications [6, 16]. Eight groups of test agents were administered to twenty two groups of adult earthworms, with each group containing three earthworms of approximately equal size. The test agents were albendazole and normal saline, serving as the positive and negative control groups, respectively, and the six crude extracts were from the leaves (LE and LTHE), seeds (SE and SHE), and stem (STE and STHE). With the exception of the negative control group (which had only one group of test organisms), all the other test agents were tested at the three concentration levels, that is, 1 mg/ml, 2.5 mg/ml, and 5.0 mg/ml (Table 1).

10 ml of each test concentration from the test agents was administered to the adult earthworms in the respective groups. Observations were made for the time taken for paralysis and death of individual worms. Paralysis was said to have occurred when the test organisms had lost mobility [20]. Similarly, death time was recorded when the test organisms were observed not to have survived, followed by fading of their body colour [20].

### 2.10. Bioassay-Guided Fractionation

Guided by the outcome of the anthelminthic effects from the crude plants, SE was selected for further investigation. The SE extract was fractionated using column chromatography with silica as the stationary phase. An optimized ratio of hexane : ethyl acetate (ratio of 4 : 1) was then adopted to achieve fractions from the SE extract, which were collected in Erlenmeyer flasks and labelled accordingly. TLC analyses were carried out on the fractions obtained using the above-mentioned solvent system. The TLC chromatogram led to the bulking of some of the fractions together based on the spot patterns observed. Five fractions were then achieved after which *in vitro* anthelmintic testing was carried out on them, to identify the most active fraction(s).

### 2.11. Statistical Analysis

The data obtained were analysed using GraphPad Prism (Prism 6 for Windows, 2012, GraphPad Software Inc., USA). The data from the evaluation of the paralysis and death times were expressed as mean ± standard error (SEM) of three earthworms in each group. Differences between means were evaluated using either the Student's *t*-test or analysis of variance (ANOVA) depending on the number of groups being compared. ANOVA analysis was usually followed by the Tukey *post hoc* test used to quantify statistical difference between specific groups. Statistical significance was established in situations with *p* values ≤ 0.05.

## 3. Results and Discussion

**Figure 3:**
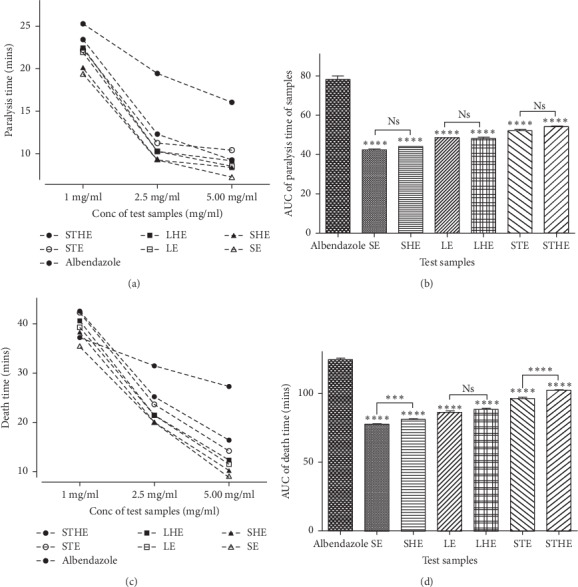
Comparing paralysis and death times from the leaves, stem bark, and seeds of *Carica papaya*. Data are presented as mean ± SEM. One-way ANOVA analysis was carried out, followed by Tukey's *post hoc* test. Plant extracts were compared with the positive control.

The phytochemical investigation showed the presence of alkaloids, saponins, and reducing sugars of glycosides present in all the crude extracts of *Carica papaya* (Table 2). Tannins were observed only in extracts of the leaves while fixed oils on the other hand were only present in the extracts of the seeds. This observation was showed to be consistent with the observations of previous studies whichreported the presence of tannins in the leaves [21, 22] and fixed oils in the seeds [23]. Some of the observations made were also not consistent with previously reported data, for example, the presence of tannins in the seeds [24] and absence of tannins in the leaves [16, 25]. The differences in the chemical composition of the test samples could be theorized to be responsible for differences in their anthelminthic effects [26], as evident in the study (Table 3 and Figure 3).

From the *in vitro* anthelminthic investigations, it was observed that the effects of the extracts on paralysis and death times were concentration dependent; increasing the concentration of the extract from 1 mg/ml to 5 mg/ml caused significant decline in both the paralysis time (*F*_(6,14)_ = 640.7, *p* < 0.0001) and death time (*F*_(6,14)_ = 1270, *p* < 0.0001) recorded (Table 3). Generally, it was observed that the paralysis and death times for the crude extracts were significantly different from each other (Figure 3). Tukey's p*ost hoc* tests showed that the recorded reduction effects for all the plant extracts on paralysis and death times were significantly higher than that from albendazole (*p* < 0.0001). This indicated that the plant parts, seeds, stem bark, and leaves of *Carica papaya*, possessed significant *in vitro* anthelminthic effects against *Pheretima posthuma*. This observation was found to be consistent with outcomes of previous studies reporting of the anthelminthic effects of seeds [7, 27, 28], stem barks and leaves [16], and the latex from the fruit [29]. It was also showed that the effects from the plant parts investigated were higher than those of the conventional anthelminthic drug, albendazole, at the concentrations adopted (*p* < 0.0001). Similar observation was made in a study involving the hydroalcoholic extracts of the leaves and stem using the same model [16]. These observations, thus, confirm and authenticate the folkloric use of the plant parts in the treatment of helminth infections [4, 14]. The similar phytocomposition for the ethanolic and hydroethanolic extracts from each plant part (Table 2) was thought to contribute to the comparable activities of such extracts in the study (*q* = 0.8651−3.861, d*f* = 14; Figure 3(b)). This was however not the case with their effects on death time, where it was shown that only extracts from the leaves were comparable (*q* = 4.392, d*f* = 14; Figure 3(d)). It was also observed that SE and SHE extracts demonstrated the highest anthelminthic effects (Figure 3). A previous study had shown that that the seeds were comparable to thiabendazole in terms of their activity against gastrointestinal helminths in Red Sokoto goats [7]. Another study had concluded that the seeds were safe and efficacious in eliminating intestinal helminths [30]. As earlier argued, this could be as a result of their peculiar chemical composition with the presence of alkaloids, saponins, glycosides, and fixed oils. Since some of these phytoconstituents have been documented to possess anthelminthic activities [31], their presence thus may contribute to the observed activity. This observation may also confirm the relative preference of the seeds to the leaves and stem bark for traditional helminth infection management [4].

**Figure 4:**
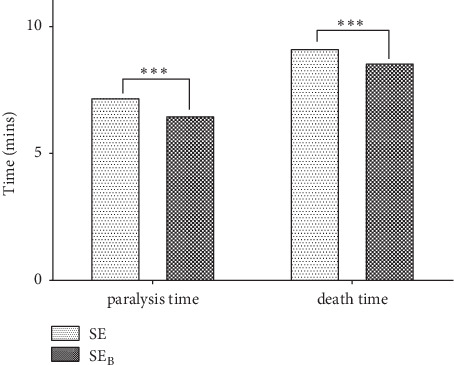
Comparing the anthelminthic effects of SE and SE_B_ extracts. Data are presented as mean ± SEM. Paired Student's *t*-test was carried out to determine difference or otherwise of the extracts.

Further fractionation of the SE extract yielded SE_B_ fraction, which also demonstrated anthelminthic effect (Table 4). It also showed that SE_B_ demonstrated a significantly higher activity than SE, both in the reduction of paralysis time (*t* = 61.49, d*f* = 2, *p*=0.0003) and death time (*t* = 98.72, d*f* = 2, *p*=0.0001) (Figure 4). Phytochemical investigation of the SE_B_ fraction showed the presence of fixed oils (Table 4). The fixed oils from the seeds have been showed to contain myristic, palmitic, stearic, arachidic, behenic, and unsaturated fatty acids among others [32], some of which possess anthelminthic effects against *Caenorhabditis elegans* [31]. It could thus be inferred that the possible presence of these compounds may account for the high activity of the fixed oils in SE_B_.

## 4. Conclusions

The results obtained from this work validate the traditional or herbal practitioners' and indigenous folks' use of *Carica papaya*, especially the seeds as an anthelmintic agent in the treatment of helminthiasis or worm infestation.

## Figures and Tables

**Table 1:** Experimental groups adopted in the study.

Test agent	Test group	Concentrations adopted	Number of test organisms in each test group
Albendazole	Positive control	1, 2.5 and 5.0 mg/ml	3
Normal saline	Negative control	—	3
Ethanolic extract from leaves	LE	1, 2.5 and 5.0 mg/ml	3
Hydroethanolic extract from leaves	LHE	1, 2.5 and 5.0 mg/ml	3
Ethanolic extract from seeds	SE	1, 2.5 and 5.0 mg/ml	3
Hydroethanolic extract from seeds	SHE	1, 2.5 and 5.0 mg/ml	3
Ethanolic extract from stem	STE	1, 2.5 and 5.0 mg/ml	3
Hydroethanolic extract from stem	STHE	1, 2.5 and 5.0 mg/ml	3

**Table 2:** Results of preliminary phytochemical investigation carried out on the crude extracts.

Crude extract	Alkaloid	Tannins	Saponins	Reducing sugars of glycosides	Fixed oils
LE	+	+	+	+	—
LHE	+	+	+	+	—
SE	+	—	+	+	+
SHE	+	—	+	+	+
STE	+	—	+	+	—
STHE	+	—	+	+	—

[ + ] present; [—] absent.

**Table 3:** Experimental results from the *in vitro* anthelminthic test on the crude extracts.

Test group	Concentration adopted	Paralysis time ± SEM (mins)	Death time ± SEM (mins)
Albendazole	1 mg/ml	25.330 ± 0.0173	37.100 ± 0.000
	2.5 mg/ml	19.450 ± 0.5751^1^	31.430 ± 0.2868^2^
	5 mg/ml	16.030 ± 0.0300^4^	27.210 ± 0.0058^4^
Normal saline	—	—	—
LE	1 mg/ml	22.030 ± 0.0058^d^	39.270 ± 0.0153^d^
	2.5 mg/ml	10.240 ± 0.0100^a,4^	21.350 ± 0.3251^a,4^
	5 mg/ml	9.120 ± 0.0200^d,4^	11.480 ± 0.2641^d,4^
LHE	1 mg/ml	22.500 ± 0.2500^a^	40.520 ± 0.2403^a^
	2.5 mg/ml	10.240 ± 0.0100^a,4^	21.380 ± 0.3101^a,3^
	5 mg/ml	8.510 ± 0.0058^d,4^	12.250 ± 0.0058^d,4^
SE	1 mg/ml	19.450 ± 0.2930^a^	35.380 ± 0.1200^a^
	2.5 mg/ml	9.300 ± 0.005^a,3^	20.030 ± 0.0115^c,4^
	5 mg/ml	7.210 ± 0.0100^d,3^	9.150 ± 0.0058^d,4^
SHE	1 mg/ml	20.163 ± 0.0318^d^	38.160 ± 0.0100^d^
	2.5 mg/ml	9.260 ± 0.0306^a,4^	20.120 ± 0.0100^c,4^
	5 mg/ml	8.310 ± 0.0100^d,4^	10.210 ± 0.0058^d,4^
STE	1 mg/ml	22.380 ± 0.3126^a^	42.120 ± 0.0058^4^
	2.5 mg/ml	11.250 ± 0.0100^a,3^	23.530 ± 0.3300^d,4^
	5 mg/ml	10.420 ± 0.2900^2,2^	14.220 ± 0.0058^d,4^
STHE	1 mg/ml	23.430 ± 0.2850^ns^	42.420 ± 0.0058^4^
	2.5 mg/ml	12.297 ± 0.0517^a,3^	25.190 ± 0.0400^a,4^
	5 mg/ml	9.280 ± 0.0200^d,4^	16.350 ± 0.0289^d,4^

[—] no observable immobility. Results are expressed as mean ± SEM. *N* = 3. Two-way ANOVA analysis, followed by Tukey's *post hoc* test was used to test for differences between paralysis and death times of plant extracts and albendazole (labelled as ^a^0.05 < *p* < 0.01; ^b^0.01 < *p* < 0.001; ^c^0.001 < *p* < 0.0001; ^d^*p* < 0.0001) and also differences among the concentrations of each plant extract (also labelled as ^1^0.05 < *p* < 0.01; ^2^0.01 < *p* < 0.001; ^3^0.001 < *p*< 0.0001; ^4^*p* < 0.0001) using 1 mg/ml as the reference, at 95% confidence level. “ns” indicated no significant difference upon comparison of groups in all cases.

**Table 4:** Outcome from bioactive fractionation from the most active crude extract (SE).

Fraction	Colour	Nature	Number of spots	Retention factor (*R*_f_)	*In vitro* activity		Phytochemical evaluation
					Paralysis time	Death time	
SE_A_	Yellow	Oily	2	0.52	—	—	
				0.79			
SE_B_	Light yellow	Oily	1	0.41	6.50 mins	8.58 mins	Fixed oils may be present
SE_C_	Deep yellow	Oily	1	0.29	—	—	
SE_D_	Orange	Oily	1	0.22	—	—	
SE_E_	Light orange	Oily	1	0.21	—	—	

[—] no observable immobility.

## Data Availability

The processed data used to support the findings of this study are included within the article.
